# Combined Plyometric and Short Sprint Training in U-15 Male Soccer Players: Effects on Measures of Jump, Speed, Change of Direction, Repeated Sprint, and Balance

**DOI:** 10.3389/fphys.2022.757663

**Published:** 2022-02-18

**Authors:** Ghaith Aloui, Souhail Hermassi, Thomas Bartels, Lawrence D. Hayes, El Ghali Bouhafs, Mohamed Souhaiel Chelly, René Schwesig

**Affiliations:** ^1^Research Unit (UR17JS01), Sport Performance, Health & Society, Higher Institute of Sport and Physical Education of Ksar Saîd, University of La Manouba, Tunis, Tunisia; ^2^Physical Education Department, College of Education, Qatar University, Doha, Qatar; ^3^Sports Clinic Halle, Center of Joint Surgery, Halle, Germany; ^4^Institute of Clinical Exercise and Health Science, School of Health and Life Sciences, University of the West of Scotland, Glasgow, United Kingdom; ^5^Department of Sports Science, Martin-Luther-University Halle-Wittenberg, Halle, Germany; ^6^Higher Institute of Sport and Physical Education of Ksar Said, University of La Manouba, Tunis, Tunisia; ^7^Department of Orthopedic and Trauma Surgery, Martin-Luther-University Halle-Wittenberg, Halle, Germany

**Keywords:** stretch-shortening cycle, short sprints training, plyometric training, training youth, soccer

## Abstract

This study examined the effect of 8 weeks of biweekly combined plyometric and short sprint training into the typical within-season training schedule of youth male soccer players. Participants were allocated at random to an experimental group (EG; *n* = 17, age: 14.6 ± 0.5 years, body mass: 60.5 ± 7.1 kg, height: 1.64 ± 0.08 m, body fat: 11.3 ± 1.4%) and a control group (CG; *n* = 17, age: 14.6 ± 0.4 years, body mass: 61.0 ± 3.9 kg, height: 1.67 ± 0.05 m, body fat: 11.8 ± 1.4%). Measures obtained pre- and post-intervention included vertical and horizontal jump performances (i.e., squat jump (SJ), countermovement jump with aimed arms (CMJA), and five-jump test (FJT)) and sprint performances (i.e., 10 and 30 m sprint). In addition, change-of-direction ability (sprint with 90° Turns (S90°) and sprint 9–3–6–3–9 m with backward and forward running (SBF)), repeated shuttle sprint ability (RSSA), and dynamic balance performance (Y balance test) were measured pre- and post-intervention. The EG experienced higher jump (all *p* < 0.05; *d* ≥ 0.71), sprint (all *p* < 0.05; *d* ≥ 0.64), change-of-direction ability (all *p* < 0.05; *d* ≥ 0.66), RSSA (all parameters except the fatigue index *p* < 0.01; *d* ≥ 0.71), and dynamic balance (all *p* ≤ 0.05; *d* ≥ 0.50) improvement compared to the CG. Adding biweekly combined plyometric and short sprint training to standard training improves the athletic performance of youth male soccer players (under 15 (U15)).

## Introduction

Competitive soccer match play is characterized by high-intensity intermittent activity patterns, whereby players are required to repeatedly sprint, turn, jump, and accelerate/decelerate which places the substantial neuromuscular load on players (Spencer et al., 2005; Stølen et al., 2005). Therefore, the ability of lower limb musculature to produce a high power output is an important fitness trait in soccer players (Wisløff et al., 2004). Speed and power are believed to be the variables predicting success in youth soccer (Reilly et al., 2000a). Specifically, sprint ability over short distances, agility performance, and vertical jumping are discriminative in terms of elite and sub-elite young soccer players (Reilly et al., 2000b). In professional soccer, various players commonly sprint over a distance between 200 and 1,100 m in a match (Rampinini et al., 2007; Andrzejewski et al., 2013). However, the average duration of sprints performed during a soccer match is typically short, ranging from 2 to 4 s (Stølen et al., 2005), with the sprint representing 1–11% of that distance (Mohr et al., 2003). Notably, 96% of sprints are less than 30 m, and 49% are less than 10 m (Stølen et al., 2005).

One specific type of power training that is easy to administer and therefore popular is plyometric jump training (PJT) (Ramirez-Campillo and Castillo, 2020; Ramirez-Campillo et al., 2021a; Sole and Ramírez-Campillo, 2021). PJT consists of exercises which take advantage of the stretch-shortening cycle (SSC) of the muscle (Komi and Gollhofer, 1999; Taube et al., 2012). Typically, plyometric jump exercises (PJT) can be performed with short (< 250 ms) or long ground contact times (> 250 ms), i.e., fast or slow SSC times (Duda, 1988; Sands et al., 2012; Faigenbaum and Chu, 2017). The utilization of the SSC enhances neural and musculotendinous ability to produce maximal force over a short duration (Wang and Zhang, 2016). Regardless of age, gender, sport, and expertise in training, plyometric training consistently results in improved vertical jumping, agility, and sprinting (Andrade et al., 2018; Ramirez-Campillo and Sanchez-Sanchez, 2020; Ramirez-Campillo et al., 2021b). In a systematic review, plyometric training increased power output in 13 of the 16 included studies, with effects ranging from 2 to 31% (Markovic and Mikulic, 2010). Furthermore, even in a short duration of less than 8 weeks, bilateral jumping coupled with unilateral drills enhanced performance (Markovic and Mikulic, 2010; Wang and Zhang, 2016).

Recently, combination training appears *en vogue* and is an efficacious tool for improving athletic performance. In this context, all of the combined sprint and plyometric (Ferley et al., 2020; Hammami et al., 2020; Kargarfard et al., 2020), agility and balance (Zemková and Hamar, 2010), plyometric and change-of-direction (Makhlouf et al., 2018; Hammami et al., 2019; Aloui et al., 2021), plyometric and sled (Prieto et al., 2021), balance and plyometric (Bouteraa et al., 2020; Guo et al., 2021), and plyometric and resistance (Almoslim, 2016; Ramirez-Campillo et al., 2018a; Fischetti et al., 2019) training programs have been effective to enhance muscle power. In addition, some recent research has indicated that combined training is superior to stand-alone approaches for increasing power and output capabilities (Chaouachi et al., 2014; Beato et al., 2018; Zghal et al., 2019; Ferley et al., 2020; Guo et al., 2021). Previous studies have recommended the continuation of combined plyometric and short sprint training into the soccer season to increase athletic performances. Hammami et al. (2020) found that such training enhanced jump performance, sprint performance, change-of-direction ability, and repeated change-of-direction ability, in youth male under 17 (U17) soccer players. Also, Sáez de Villarreal et al. (2015) reported significant increases in jump performance, sprint performance, and change-of-direction ability following combined plyometric-speed training and technical drills work, in youth male under 15 (U15) soccer players.

In fact, combined plyometric and short sprints seem to be the least scientifically investigated combination training mode than the others mentioned previously such as plyometrics and resistance, plyometrics and sled, and balance and plyometrics. To our knowledge, despite the above literature, there is a paucity of data concerning the efficacy of combined plyometric and short sprints on physical performance in youth male soccer players. Therefore, this study evaluated the effects of replacing normal training during the season with combined plyometric and short sprint training in elite male U15 soccer players. Herein, we examined vertical and horizontal jump performances (i.e., squat jump (SJ), countermovement jump with aimed arms (CMJA), five-jump test (FJT)), sprint performance (i.e., 10 and 30 m sprint), change-of-direction ability (sprint with 90° Turns (S90°) and sprint 9–3–6–3–9 m with backward and forward running (SBF)), repeated shuttle sprint ability (RSSA), and dynamic balance performance (Y balance test).

We hypothesized that replacing some of the regular in-season training with a biweekly intervention of this type would increase both horizontal and vertical jump performances, sprint performance, and the change-of-direction ability after 8 weeks.

## Materials and Methods

### Participants

Thirty-four participants of all positions from a single male soccer team in the National First Division, with a playing experience of 4.4 ± 0.8 years, were participated. Prior to participation, participants were deemed fit to participate in the study, without orthopedic or other conditions which would preclude them from undertaking the plyometric and change-of-direction training. Subjects were randomly allocated to an experimental group (EG; *n* = 17, age: 14.6 ± 0.5 years, body mass: 60.5 ± 7.1 kg, height: 1.64 ± 0.08 m, body fat: 11.3 ± 1.4%) and a control group (CG; *n* = 17, age: 14.6 ± 0.4 years, body mass: 61.0 ± 3.9 kg, height: 1.67 ± 0.05 m, body fat: 11.8 ± 1.4%). There was no baseline difference between the groups in anthropometric characteristics (*p* ≥ 0.05). Participants were without any problems regarding health status and physical condition as they had completed a 6-week preseason training period of 5-6 sessions each week.

### Experimental Design

Both groups undertook five training sessions per week (∼90 min each session), plus a competitive match one time per week. Conditioning training was completed two times per week, with the first session developing aerobic fitness through small-sided games while the second session targeted anaerobic fitness with resistance (40-60% 1 RM). The controls maintained their normal training schedule throughout the 2 months of the intervention, whereas the EG replaced the technical-tactical part of their standard regimen by combined plyometric and short sprint training. Participants also engaged in the 60-min school physical education sessions weekly (Table 1).

**TABLE 1:** Details of general training routine during the week performed by both control and experimental group over the 8-weeks intervention.

Days	Objectives
Mondays	Rest
Tuesdays	Aerobic capacity training and defensive tactics training
Wednesdays	Aerobic power training and defensive tactics training
Thursdays	Power anaerobic training and defensive and offensive tactics training
Fridays	Technical training and offensive tactics training
Saturdays	Technical training and offensive tactics training
Sunday	Official matches

The University Institutional Review Committee for the ethical human experimentation approved the interventional study (pre- and post-design, CG vs. EG), which was in line with current national laws and regulations (reference number: KS000002020; date of approval: November 10, 2020). Participants (and their guardians, in the case of minors) gave written informed consent. Two familiarization sessions preceded the testing period by 2 weeks, which was 2 months into the competitive season.

### Combined Plyometric and Short Sprint Training

**FIGURE 1:**
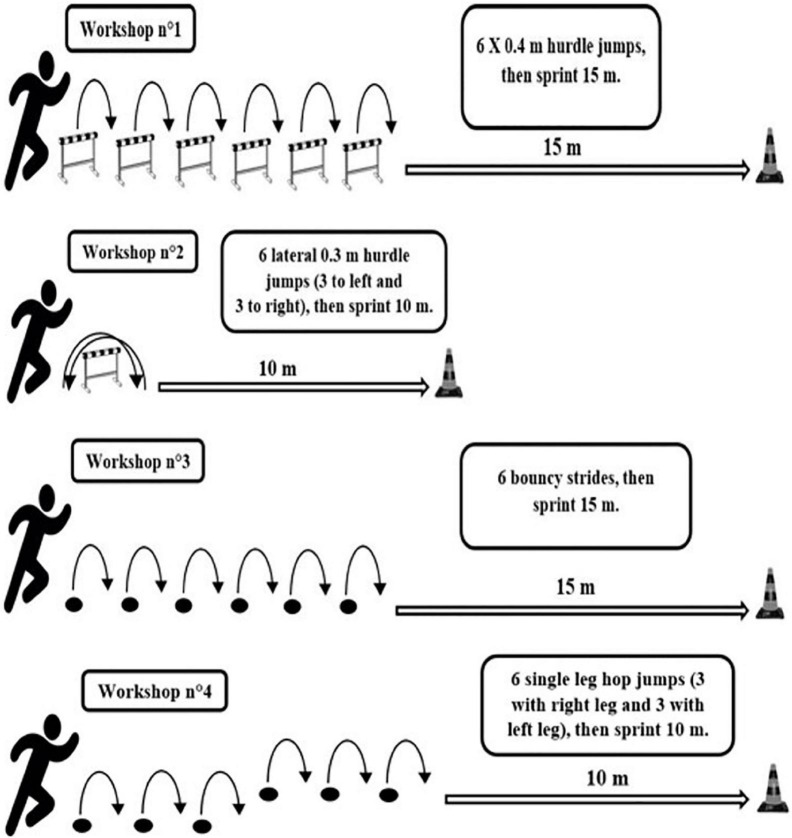
Exercise used in the combined plyometric training program.

The training group undertook four sessions or workshops, biweekly (Figure 1). Workshops commenced with plyometric exercises (i.e., hurdle jumps, lateral hurdle jumps, bouncy strides, and single-leg hop jumps) and ended with a short sprint of 10-15 m (Table 2). The total number of ground contacts per session was gradually increased throughout the intervention (from 72 to 144 ground contacts per session), as well as the number of sets (from 3 to 6 sets) for each workshop. The number of contacts per set was maintained at 6 contacts. A 90-s rest interval was planned between each set of exercises to allow sufficient recovery time (Negra et al., 2016). Jump training protocols were supervised by a qualified instructor. The training protocol was based on previously published recommendations for training volume and intensity from the study by Bedoya et al. (2015). Furthermore, the sprint training protocol was based on previously published recommendations for sprint distances (Sáez de Villarreal et al., 2015; Kargarfard et al., 2020).

**TABLE 2:** Plyometric components introduced into the program of the experimental group.

Week	Workshop 1	Workshop 2	Workshop 3	Workshop 4	Total (contact)
1	3 Repetitions	3 Repetitions	3 Repetitions	3 Repetitions	72
2	3 Repetitions	3 Repetitions	3 Repetitions	3 Repetitions	72
3	4 Repetitions	4 Repetitions	4 Repetitions	4 Repetitions	96
4	4 Repetitions	4 Repetitions	4 Repetitions	4 Repetitions	96
5	5 Repetitions	5 Repetitions	5 Repetitions	5 Repetitions	120
6	5 Repetitions	5 Repetitions	5 Repetitions	5 Repetitions	120
7	6 Repetitions	6 Repetitions	6 Repetitions	6 Repetitions	144
8	6 Repetitions	6 Repetitions	6 Repetitions	6 Repetitions	144

### Testing Schedule

Tests were conducted at least 3 days after the last competitive match and 5-9 days after the previous training session. Testing took place on a tartan surface integrated into the weekly training schedule of players and a standardized warm-up preceded each test. Tests were conducted on three separate days in the following order:

• Day 1: anthropometric assessment, SJ, CMJA, S90°;

• Day 2: Y balance test, 10 and 30 m sprint, SBF;

• Day 3: FJT and RSSA.

The 10 and 30 m sprint performance, S90°, SBF, SJ, CMJA, FJT, Y balance test, and the RSSA have all been previously described in detail (Sporis et al., 2010; Hammami et al., 2020; Negra et al., 2020) so are not repeated in this study to avoid self-plagiarism and for brevity. Anthropometric characteristics (body mass and body fat percentage) were evaluated barefoot in the morning after an overnight fast, with bioelectrical impedance analysis (BIA) (BC-602, Tanita Co., Tokyo, Japan; Sitko et al., 2020). Each test was explained and demonstrated by one of the trained assessors before the athlete started practice trials. The same assessor supervised the same test station during pre- and posttest to ensure consistency. A 90-s rest interval was planned between each set of exercises to allow sufficient recovery time (Negra et al., 2016).

### Statistical Analyses

All statistical analysis was conducted using SPSS version 28.0 for Windows (IBM, Armonk, NY, United States). Normal distribution was tested using the Shapiro–Wilk test. The results showed that the majority of parameters showed normal distribution (76%). However, 24% (9/37) of the other parameters are not normally distributed (body fat: *p* = 0.023, SJ 1: *p* = 0.017, SJ 2: *p* = 0.006, CMJA 1: *p* = 0.002, FJT 1: *p* = 0.001, FJT 2: *p* = 0.039, repeated sprint ability (RSA) fatigue index 1: *p* = 0.016, anterior direction of left leg 2: *p* = 0.010, and posteromedial direction of left leg 2: *p* = 0.034). Homogeneity of variance was determined using the Levene’s test. Notably, 38% (6/16) of variance tests indicated a missing of homogeneity of variance (SJ: *p* = 0.012, sprint 10 m: *p* = 0.038, RSA best time: *p* = 0.032, RSA mean time: *p* = 0.042, RSA fatigue index: *p* < 0.001, and posteromedial direction of left leg: *p* = 0.027).

A sample size calculation (nQuery Advisor 4.0; Statistical Solutions, Saugus, MA, USA) provided a sample size with *n* = 7 in each group. The calculation is based on a mean difference of 0.4 s in 10 m sprint using a two-sided *t*-test with the α level of 0.05 and a pooled SD of 0.3 s, with a statistical power of 0.8 (Bortz, 1999).

Independent samples *t*-tests examined baseline intergroup differences. Paired sample *t*-tests tested for pre-to-post performance changes within each group. Training effects were assessed by mixed factorial 2-way (group × time) ANOVA with repeated measures. Subsequently, Tukey’s *post-hoc* tests examined pairwise differences. Alpha levels are reported as exact *p*-values as suggested by the American Statistical Association (Hurlbert et al., 2019). Effect sizes are reported as Cohen’s *d* and are interpreted as trivial (< 0.20), small (≥ 0.20–0.49), moderate (≥ 0.50–0.79), and large (≥ 0.80) (Cohen, 1988). Percentage changes were calculated as ((post-training value − pre-training value)/pre-training value) × 100. Reliability was assessed using intraclass correlation coefficients (ICCs) (Vincent, 1995) and the coefficients of variation (CVs) over consecutive pairs of intra-participant trials (Schabort et al., 1998). Vertical and horizontal jump parameters, sprint parameters, change-of-direction ability, and balance performance had an ICC > 0.80 and a CV < 5% (Table 3). Data are reported as mean ± SD.

**TABLE 3:** Interclass correlation coefficient (ICC, 95% confidence intervals (CI)) and coefficient of variation (CV) for all performance tests.

	ICC (95% CI)	CV (95%CI) [%]
***Sprint times***		
10 m (s)	**0.95** (0.91-0.98)	**1.6** (1.2-2.1)
30 m (s)	**0.93** (0.89-0.97)	**1.9** (1.6-2.3)
**Change of direction test**		
S90° (s)	**0.93** (0.90-0.96)	**1.7** (1.4-2.2)
SBF (s)	**0.91** (0.88-0.95)	**1.8** (1.5-2.3)
***Vertical jump***		
SJ (cm)	**0.94** (0.90-0.98)	**2.0** (1.6-2.5)
CMJA (cm)	**0.92** (0.88-0.96)	**2.2** (1.8-2.7)
***Horizontal jump***		
FJT (m)	**0.84** (0.80-0.88)	**4.1** (3.6-4.5)
***Y-balance test***		
**Right support leg**		
Anterior direction (cm)	**0.96** (0.91-0.99)	**4.5** (4.0-4.9)
Posteromedial direction (cm)	**0.95** (0.91-0.98)	**4.8** (4.0-5.2)
Posterolateral direction (cm)	**0.93** (0.90-0.98)	**4.8** (4.0-5.3)
**Left support leg**		
Anterior direction (cm)	**0.95** (0.90-0.98)	**4.6** (4.1-5.1)
Posteromedial direction (cm)	**0.93** (0.89-0.98)	**4.8** (4.2-5.3)
Posterolateral direction (cm)	**0.92** (0.88-0.97)	**4.7** (4.1-5.2)

*ICC > 0.75 and CV < 10% marked in bold.*

## Results

Reliability is summarized in Table 3. ICC and CV, including 95% CI, show acceptable reliability for all variables. No parameter showed between-group differences at baseline.

### Training Effects on Jump Performance

There were interaction effects (group × time) for vertical and horizontal jump performances, with the EG outperforming the CG in terms of improvement from pre- to post-intervention (SJ: Δ19%, *p* < 0.01, *d* = 0.71; CMJA: Δ20%, *p* < 0.05, *d* = 0.86; FJT: Δ15%, *p* < 0.05, *d* = 0.74; Table 4).

**TABLE 4:** Vertical and horizontal jump test, sprint times, and change of direction performance performances in experimental and control group before and after 8-weeks intervention.

	Experimental (*n* = 17)			Paired *t*-test		Control (*n* = 17)			Paired *t*-test		ANOVA (group x time)	
	Pre	Post	% Δ	P	ES	Pre	Post	% Δ	*P*	ES	*p*	ES
**Vertical jump**												
SJ (cm)	24.5 ± 3.13	29.1 ± 3.36	19.1 ± 2.03	< 0.001	1.43	24.5 ± 2.00	25.4 ± 2.09	3.74 ± 1.19	< 0.001	0.45	0.006	0.71 (medium)
CMJA (cm)	30.8 ± 2.93	36.9 ± 3.14	20.0 ± 2.64	< 0.001	2.02	30.7 ± 2.98	31.9 ± 2.90	3.82 ± 0.99	< 0.001	0.39	0.001	0.86 (large)
**Horizontal jump**												
FJT (m)	9.33 ± 0.63	10.7 ± 0.64	15.0 ± 1.49	< 0.001	2.20	9.53 ± 0.68	10.0 ± 0.65	4.93 ± 1.44	< 0.001	0.70	0.004	0.74 (medium)
**Sprint**												
10 m (s)	2.07 ± 0.10	1.84 ± 0.08	−11.1 ± 1.60	< 0.001	2.51	2.03 ± 0.16	1.98 ± 0.15	−2.64 ± 0.56	< 0.001	0.35	0.006	0.71 (medium)
30 m (s)	5.23 ± 0.34	4.75 ± 0.28	−8.98 ± 1.24	< 0.001	1.51	5.19 ± 0.28	5.08 ± 0.26	−2.07 ± 0.48	< 0.001	0.40	0.012	0.64 (medium)
**Change of direction Performance**												
S90° (s)	7.67 ± 0.33	7.00 ± 0.28	−8.65 ± 0.85	< 0.001	2.18	7.71 ± 0.32	7.50 ± 0.30	−2.69 ± 0.61	< 0.001	0.67	0.003	0.76 (medium)
SBF (s)	9.12 ± 0.35	8.38 ± 0.36	−8.14 ± 0.68	< 0.001	2.08	9.10 ± 0.41	8.85 ± 0.40	−2.79 ± 0.66	< 0.001	0.63	0.011	0.66 (medium)

### Training Effects on Sprint Performance

There was a training effect (group × time) on sprint performance, with the EG improving more than CG (10 m sprint: Δ−11%, *p* < 0.01, *d* = 0.71; 30 m sprint: Δ−9%, *p* < 0.05, *d* = 0.64; Table 4).

### Training Effects on Change-of-Direction Ability

There was a training effect for S90° and SBF with the EG improving more than CG (S90°: Δ−9%, *p* < 0.01, *d* = 0.76; SBF: Δ−8%, *p* < 0.05, *d* = 0.66; Table 4).

### Training Effects on Repeated Shuttle Sprint Ability

For the ability to perform RSS, the RSSA test also showed group × time interactions for most of its parameters (RSSA best and RSSA mean), with time decreases of Δ−8% (*p* < 0.01, *d* = 0.72) and Δ−8% (*p* < 0.01, *d* = 0.71; Table 5).

**TABLE 5:** Repeated sprint shuttle ability test performances and Y-balance test performances in experimental and control group before and after 8-weeks intervention.

	Experimental (*n* = 17)			Paired *t*-test		Control (*n* = 17)			Paired *t*-test		ANOVA (group x time)	
	Pre	Post	% Δ	*p*	ES	Pre	Post	% Δ	*p*	ES	P	ES
**Repeated Shuttle Sprint Ability parameters**												
Fastest time (s)	8.30 ± 0.41	7.63 ± 0.40	−8.07 ± 0.71	< 0.001	1.64	8.32 ± 0.22	8.10 ± 0.24	−2.57 ± 0.53	< 0.001	0.93	0.006	0.72 (medium)
Mean time (s)	8.45 ± 0.43	7.76 ± 0.41	−8.10 ± 0.68	< 0.001	1.64	8.42 ± 0.23	8.21 ± 0.24	−2.56 ± 0.56	< 0.001	0.93	0.006	0.71 (medium)
Fatigue index (%)	1.76 ± 0.55	1.72 ± 0.48	0.01 ± 9.51	0.408	0.63	1.28 ± 0.24	1.28 ± 0.25	0.85 ± 9.30	0.844	0.02	0.848	0.06 (small)
**Y-balance test - Right support leg**												
Anterior direction (cm)	82.5 ± 7.67	92.2 ± 8.07	10.5 ± 1.00	< 0.001	1.22	85.2 ± 6.39	87.9 ± 7.95	3.03 ± 0.63	< 0.001	0.42	0.048	0.50 (medium)
Posteromedial direction (cm)	101 ± 7.73	112 ± 7.79	9.83 ± 1.42	< 0.001	1.43	105 ± 6.45	108 ± 6.34	2.73 ± 0.80	< 0.001	0.46	0.022	0.59 (medium)
Posterolateral direction (cm)	55.2 ± 7.54	65.3 ± 8.43	15.6 ± 1.05	< 0.001	1.26	56.9 ± 7.52	58.7 ± 7.78	3.10 ± 0.80	< 0.001	0.24	0.033	0.54 (medium)
**Y-balance test - Left support leg**												
Anterior direction (cm)	86.1 ± 6.60	96.4 ± 8.16	10.6 ± 1.50	< 0.001	1.39	87.7 ± 7.70	90.1 ± 7.94	2.67 ± 0.89	< 0.001	0.31	0.037	0.53 (medium)
Posteromedial direction (cm)	106 ± 8.64	118 ± 8.33	9.87 ± 2.10	< 0.001	1.37	101 ± 5.38	104 ± 5.30	2.43 ± 1.17	< 0.001	0.47	0.011	0.66 (medium)
Posterolateral direction (cm)	55.4 ± 6.38	65.1 ± 7.11	14.9 ± 0.88	< 0.001	1.43	56.5 ± 7.95	58.4 ± 8.18	3.24 ± 0.94	< 0.001	0.23	0.035	0.54 (medium)

### Training Effects on Balance Performance

There was a training effect on Y balance test performance with the EG improving more for balance measured on both legs (anterior: Δ11%, *p* = 0.05, *d* = 0.50; posteromedial: Δ10%, *p* < 0.05, *d* = 0.59; posterolateral: Δ16%, *p* < 0.05, *d* = 0.54 for right support leg). For the left support leg, we found similar results (anterior: Δ11%, *p* < 0.05, *d* = 0.53; posteromedial: Δ10%, *p* < 0.05, *d* = 0.66; posterolateral: Δ15%, *p* < 0.05, *d* = 0.54; Table 5).

## Discussion

This study tested the efficacy of an 8-week combined plyometric and short sprint training program at improving measures of athletic performance in elite adolescent soccer players. We observed that replacing some aspects of typical training with combined plyometric and short sprint training improved vertical and horizontal jump performances, sprinting, change-of-direction ability, the ability to RSS, and balance.

### Effect of Training on Jump Performance

The ability to jump is a distinctive feature of success in adult and young soccer players (Söhnlein et al., 2014; Ramirez-Campillo et al., 2018b). Therefore, developing the ability of a soccer player to generate vertical power quickly is likely to be advantageous for the competition (Oliver et al., 2021). The results of this study suggest the intervention effects on vertical and horizontal jump performances (Table 4). Our findings are confirmed by Hammami et al. (2020) who observed improvements in vertical and horizontal jump performances following combined plyometric and short sprint training in male U17 soccer players. In addition, Sáez de Villarreal et al. (2015) reported significant increases in vertical jump performance following combined plyometric-speed training and technical drills work, in male youth soccer players (U15). Jump performance augmentation could be the result of neuromuscular adaptations such as greater neural drive of agonist muscles, changes in mechanical stiffness of tendons, size changes and/or the architecture of muscles, and changes in the mechanics of single fibers (Maffiuletti et al., 2002; de Villarreal et al., 2009; Thomas et al., 2009). Other potential mechanisms include (i) changes in muscle activation strategies (or intermuscular coordination) during vertical jumps, especially during the preparatory phase jump; and (ii) changes in the stretch reflex excitability (Bishop and Spencer, 2004; de Villarreal et al., 2009).

### Effect of Training on Sprint Performance

Stølen et al. (2005) reported that 96% of sprint bouts during soccer match play are shorter than 30 m, with 49% being shorter than 10 m. The present results showed significant intervention effects in the EG compared to the CG for 10 and 30 m sprint performance. This corroborates findings reported by Sáez de Villarreal et al. (2015), who examined the efficacy of combined plyometric-speed training and technical drills work in youth male soccer players (U15) and noted improved sprint performance over 5 and 10 m. Moreover, Kargarfard et al. (2020) investigated the effects of combined plyometric and short sprint exercises in youth male under 19 (U19) soccer players. They reported improved sprint performance over 30 m. The improved sprint performance observed in this study may be useful for soccer players as it would correspond to an advantage in duels sprints, which can allow players to reach the ball before the opponent (Stølen et al., 2005).

### Effect of Training on Change-of-Direction Ability

During soccer match play, players perform many accelerations and sprints at maximum speed (Haugen et al., 2014), with and without changes in direction (Bloomfield et al., 2007; Barnes et al., 2014). The present investigation demonstrated the intervention effects on the change-of-direction ability. There are few studies evaluating the effectiveness of combined plyometric and short sprint training program on change-of-direction ability, in soccer players (Sáez de Villarreal et al., 2015; Hammami et al., 2020; Kargarfard et al., 2020). This study corroborates the results obtained by Sáez de Villarreal et al. (2015), who studied the effects of combined plyometric-speed training and technical drills work in youth male soccer players (U15). They noted improvements in change-of-direction ability. Indeed, Kargarfard et al. (2020) observed significant increases in change-of-direction ability, following combined plyometric and short sprint training, in youth male soccer players (U19). Improvements in the change-of-direction ability necessitate rapid development of strength, eccentric force production in the lower limbs, and a rapid change from eccentric muscle to concentric contraction in the knee extensors. Plyometric training can enhance these factors (Miller et al., 2006; Sheppard and Young, 2006).

### Effect of Training on Repeated Shuttle Sprint Ability

The present study demonstrated intervention effects on all RSSA parameters except the fatigue index. Our results are in line with those of Hammami et al. (2020) who reported improvement in all repeated change-of-direction ability parameters except the fatigue index in male U17 soccer players following combined plyometric and short sprint training. In contrast, our results contradict those reported by Kargarfard et al. (2020) who reported no improvements in RSA following combined plyometric and short sprint training in male U19 soccer players. Improvements in RSA may correspond to increased running efficiency (Balsalobre-Fernández et al., 2016) or increased tendon stiffness (Markovic and Mikulic, 2010), which allows a faster transfer of force from the contracting muscles, reducing reaction times, and improving change-of-direction ability (Markovic and Mikulic, 2010; Balsalobre-Fernández et al., 2016). Although maximum sprint speed is one of the performance composites of anaerobic performance in repeated sprints (Girard et al., 2011), other factors determining inter-sprint recovery and maintenance of performance are required to enhance RSA (da Silva et al., 2010; Girard et al., 2011).

### Effect of Training on Balance Performance

Chimera et al. (2004) reported that incorporating plyometric exercises into athlete training reduces the risk of injury by improving the functional stability of the leg joints. Our study seems to be the first to examine the effects of the combined plyometric and short sprint training program on balance performance, in male U15 soccer players. Our results of improved balance disagree with those obtained by Hammami et al. (2020) who examined the efficacy of combined plyometric and short sprints, in male U17 soccer players and reported no improvements in static and dynamic balance performance. Differences in the combined plyometric and short sprint training program (duration, intensity, frequency, and type of exercise) and in methodology (age group and competitive level of the study population; duration of intervention) could contribute to these discrepancies. Improvements in balance reduced the risk of lower limb injury in soccer players (Lloyd, 2001). In the context of increased athletic performance (Zech et al., 2010), data from the present investigation emphasize the importance of plyometric training as an effective injury-reduction strategy in young athletes. A systematic review (Hübscher et al., 2010) showed evidence of the effectiveness of balance training in reducing the incidence of certain types of sports injuries among adolescent and young athletes during pivoting sports. Recently, Rivera et al. (2017) reported that balance training programs were effective in reducing the incidence rates of ankle sprains, including those with and those without a history of ankle sprains, in soccer players. In addition, the increased balance may be related to an improvement in lower extremity muscle co-contraction (Lloyd, 2001) or the changes in proprioception and neuromuscular control (Michailidis et al., 2019).

## Conclusion

Notably, the 8-week, biweekly, in-season, combined plyometric and short sprint training improves strength and balance performance in elite youth soccer players (U15). Therefore, male youth soccer players may benefit from combined plyometric and short sprint training during the competitive season and yield positive results in the performance measures. Practically speaking, the total training time was only 15–30 min per session, which can be incorporated into a habitual soccer training two times per week.

Limitations of this study are the relatively small sample size, although this is consistent with a squad size of this age and ability, and those results are applicable to one particular category of youth soccer players and cannot be extrapolated to other sexes or age groups. In future studies, plyometric or sprint training should be examined separately and in combination to study the effect of these programs on different athletic performances in young soccer players. It is important to determine what exactly (i.e., plyometrics, sprinting, or combination) improves performance. However, this would necessitate considerably greater resource commitment. Further research is needed in other age groups, female soccer players, and other skill levels to verify the effectiveness of combined plyometric and short sprint training.

## Data Availability Statement

The original contributions presented in the study are included in the article/supplementary material, further inquiries can be directed to the corresponding author/s.

## Ethics Statement

The studies involving human participants were reviewed and approved by Review Board of Research Unit (UR17JS01) Sport Performance, Health & Society, Higher Institute of Sport and Physical Education, Ksar-Saîd, University. Written informed consent to participate in this study was provided by the participants’ legal guardian/next of kin.

## Author Contributions

GA and MC contributed to conceptualization. GA contributed to methodology, software, formal analysis, investigation, data curation, writing—original draft preparation, and project administration. GA, MC, and TB contributed to validation. MC contributed to resources and supervision. SH and LH contributed to writing—review and editing. EB contributed to visualization. RS contributed to funding acquisition. All authors have read and agreed to the published version of the manuscript.

## Conflict of Interest

The authors declare that the research was conducted in the absence of any commercial or financial relationships that could be construed as a potential conflict of interest.

## Publisher’s Note

All claims expressed in this article are solely those of the authors and do not necessarily represent those of their affiliated organizations, or those of the publisher, the editors and the reviewers. Any product that may be evaluated in this article, or claim that may be made by its manufacturer, is not guaranteed or endorsed by the publisher.
